# Global Fit Analysis of Myosin-5b Motility Reveals Thermodynamics of Mg^2+^-Sensitive Acto-Myosin-ADP States

**DOI:** 10.1371/journal.pone.0064797

**Published:** 2013-05-23

**Authors:** Igor Chizhov, Falk K. Hartmann, Nikolas Hundt, Georgios Tsiavaliaris

**Affiliations:** Institute for Biophysical Chemistry, OE 4350, Hannover Medical School, Hannover, Germany; University of Heidelberg Medical School, Germany

## Abstract

Kinetic and thermodynamic studies of the mechanochemical cycle of myosin motors are essential for understanding the mechanism of energy conversion. Here, we report our investigation of temperature and free Mg^2+^-ion dependencies of sliding velocities of a high duty ratio class-5 myosin motor, myosin-5b from *D. discoideum* using *in vitro* motility assays. Previous studies have shown that the sliding velocity of class-5 myosins obeys modulation by free Mg^2+^-ions. Free Mg^2+^-ions affect ADP release kinetics and the dwell time of actin-attached states. The latter determines the maximal velocity of actin translocation in the sliding filament assay. We measured the temperature dependence of sliding velocity in the range from 5 to 55°C at two limiting free Mg^2+^-ion concentrations. Arrhenius plots demonstrated non-linear behavior. Based on this observation we propose a kinetic model, which explains both sensitivity towards free Mg^2+^-ions and non-linearity of the temperature dependence of sliding velocity. According to this model, velocity is represented as a simple analytical function of temperature and free Mg^2+^-ion concentrations. This function has been applied to global non-linear fit analysis of three data sets including temperature and magnesium (at 20°C) dependence of sliding velocity. As a result we obtain thermodynamic parameters (ΔH_Mg_ and ΔS_Mg_) of a fast equilibrium between magnesium free (AM**·**D) and magnesium bound acto-myosin-ADP (AM**·** Mg^2+^D) states and the corresponding enthalpic barriers associated with ADP release (ΔH_1_^‡^ and ΔH_2_^‡^). The herein presented integrative approach of data analysis based on global fitting can be applied to the remaining steps of the acto-myosin ATPase cycle facilitating the determination of energetic parameters and thermodynamics of acto-myosin interactions.

## Introduction

An intrinsic property of myosins is the ability to convert chemical energy from ATP hydrolysis into mechanical force and movement through the cyclic interaction with actin filaments. Despite the high degree of structural conservation of the myosin motor domains [1] and a well defined biochemical ATPase cycle shared by all types of myosins investigated so far [2], the motors display a large variety of mechanochemical activities that range from the generation of force and tension in contractile processes e.g. muscle contraction, cellular movement, or cytokinesis [3] to strain sensing and intracellular transport functions of proteins and organelles [4]. Kinetic studies have revealed that variations in (*i*) the maximal actin-activated ATPase activities, (*ii*) the rate constants and equilibria of their interactions with actin and nucleotides, (*iii*) number and degree of population of the individual states of the ATPase cycle, and (*iv*) the fraction of the total cycle time the motors spent in each state, contribute to their different modes of mechanical activities [5]. External strain, load, and cooperative effects are additional factors that influence the motor properties of myosins [6], [7], [8], [9].

One important parameter that varies between the myosins is the fraction of time the motors spend in the strongly actin attached states during the ATPase cycle [10]. This parameter termed duty ratio classifies the myosins in high and low duty ratio motors [11]. Fast myosins such as muscle myosin-2 have a low duty ratio, while processive class-5 myosins display a high duty ratio. Biochemical studies correlate changes of the duty ratio with the rates of transition and isomerization states associated with the release of the hydrolysis products, including the dissociation of Mg^2+^ from the nucleotide binding pocket [12], [13]. In myosin-2, the actin-accelerated rate-limiting release of P_i_ precedes the fast dissociation of Mg^2+^ADP [14]; whereas in myosin-5 the dwell time of attached state is determined by the rate of ADP release [15]. In addition kinetic investigations revealed an equilibrium between magnesium free (AM**·**D) and magnesium bound acto-myosin-ADP (AM**·**Mg^2+^D) states [16], [17], [18]. Therefore, the fraction of time the myosin remains attached to actin can be affected by this equilibrium with consequences for processivity and motile activity [19], [20], [21]. While the kinetic properties and the mechanism of myosin-5 movement are well established [22], [23], [24], only little information regarding the energetics of the individual steps in the ATPase cycle of the motor is available [25]. An ultimate goal for a complete and detailed description of the myosin-5 mechanism of energy transduction should thus include the determination of the energetics of the individual steps in the acto-myosin ATPase cycle. Following the changes of rates and equilibria at different thermodynamic conditions e.g. by varying the temperature, important information on enthalpies and entropies of the intermediates that emerge during the enzymatic cycle of the acto-myosin interaction can be obtained.

Previous thermodynamic investigations have addressed temperature dependences of individual myosins in terms of their ATPases, motile activities, or ADP-release and nucleotide induced acto-myosin dissociation kinetics [25], [26], [27]. In the current study we extended the analysis of the myosin-5 ATPase cycle to thermodynamic experiments using *in vitro* motility assays over an extended temperature range with a high duty ratio class-5 myosin from *Dictyostelium*. Myosin-5b is a processive motor like myosin-5a and possesses motor properties that can be modulated by changes in the concentration of free Mg^2+^-ions [20]. Global fit analysis of the temperature and magnesium dependent velocities using a simple two-state kinetic model allowed us to describe the thermodynamics of two essential Mg^2+^-sensitive ADP release steps in the ATPase cycle of myosin-5b. Our results provide important insights on the critical role of the 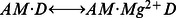 equilibrium for the high duty ratio of myosin-5 motors.

## Materials and Methods

### Reagents and proteins

Standard chemicals, anti-His antibodies, trichloromethyl-silane (TCMS), and tetramethyl rhodamine isothiocyanate (TRITC)-phalloidin were purchased from Sigma; rabbit skeletal muscle actin was purified from acetone powder and labeled with TRITC-phalloidin. A titrated 3.9 M stock solution of MgCl_2_ purchased from Sigma was used for the adjustment of free Mg^2+^-ion concentrations in the experimental buffers. *D. discoideum* myosin-5b motor domain fused to an artificial lever arm consisting of two α-actinin repeats (2R) and C-terminal octa-His tag was purified according to the previously described procedures [20].

### *In vitro* motility assays

All experiments were performed in assay buffer (AB) containing 25 mM imidazole pH 7.4, 25 mM KCl, 1 mM EGTA, 10 mM DTT. Preparation of actin and labelling with TRITC phalloidin was done according to [28]. The concentration of ATP was 4 mM and the concentration of MgCl_2_ was adjusted accordingly to provide the desirable free Mg^2+^-ion concentration using the Maxchelator software (http://maxchelator.stanford.edu). Free Mg^2+^-ion concentrations are assigned as [Mg^2+^]. The affinity of Mg^2+^ for ATP is temperature dependent in aqueous solution and changes in the studied temperature range from approximately 140 µM (5°C) to 50 µM (55°C). We ignored this temperature effect for the calculation of free Mg^2+^-ions concentrations in the assay buffer, since we used excess ATP concentrations making the error of free Mg^2+^-ions concentrations negligibly small in the temperature range studied. Saturating concentrations of myosin molecules were used to obtain maximum sliding velocities. The myosin molecules were immobilized via anti-penta-His antibodies (concentration 0.025 mg/ml) on silanated glass surfaces of a flow-cell. TCMS coated cover slips were prepared according to [29]. Actin sliding motility was recorded at temperatures ranging from 5°C to 55°C using an Olympus IX81 inverted fluorescence microscope (Olympus, Hamburg, Germany) as described previously [20]. For temperature control, a spectroscopic flow-through cuvette (Type 137-QS, Hellma Analytics GmbH) was attached to the upper surface of the cover slip using silicon grease. The temperature was changed and adjusted with a water-thermostat connected to the flow-through cuvette. For temperatures above 20°C the microscope was additionally heated using objective and stage heaters. A thermal-couple digital thermometer (Center 301, Center Technology Corp., Taiwan) was directly attached to the contact between cuvette and cover slip providing reliable control of the sample temperature. The movement of more than 200 TRITC-phalloidin labeled actin filaments was recorded for each temperature point. Three independent measurements have been used for data analysis.

### Data analysis

Actin filament tracking was performed with DiaTrack 3.01 (Semasopht, Switzerland). Data analysis and graphical representation of results were done with Origin 7.0 (OriginLab Corporation, U.S.A.). The non-linear least square fitter of Origin 7.0 was used for global data analysis. Equation 1 was implemented as user defined function to the fit. Global fit included three experimental data sets, each sharing the same six parameters (*v*_1_, *v*_2_, ΔH_1_^‡^, ΔH_2_^‡^, ΔH_Mg_, ΔS_Mg_), which are assumed to be temperature independent. The function was written in the script form of the software to allow conditional reassignment of the independent variables, either as reciprocal temperature or concentration of free Mg^2+^-ions. Additionally, errors of experimental data were included to the fit as statistical weighting parameters. The global fit analysis was performed several times with different initial guesses of parameters to avoid solutions which could correspond to local minima of fit.

## Results and Discussion

**Figure 1:**
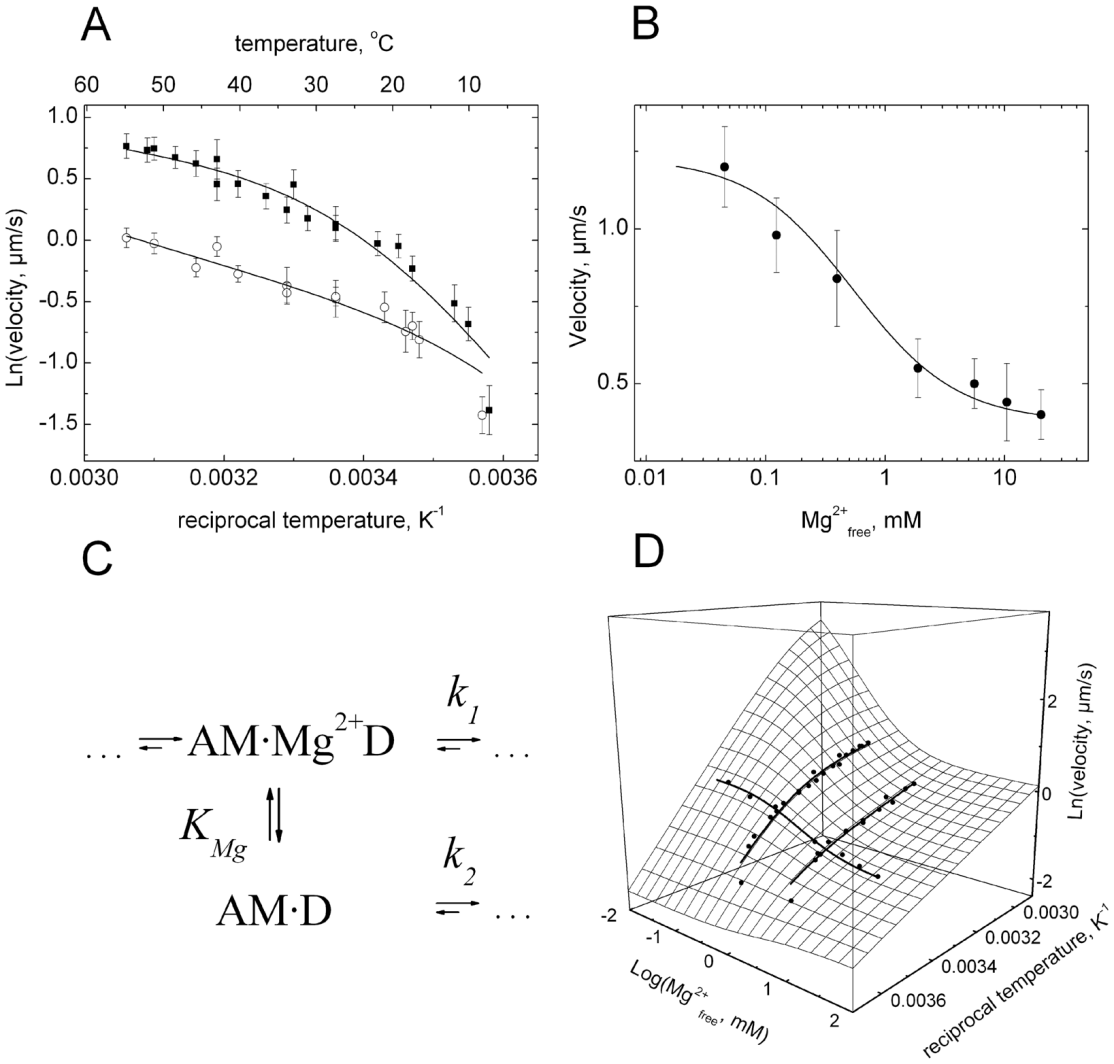
Dependence of myosin-5b sliding velocity on reciprocal Kelvin temperature (A) and free Mg^2+^ ion concentration (B). The data in panel A show sliding velocities at two limiting Mg^2+^ ion concentrations of 4.5 mM (open circles) and 0.05 mM (solid squares) over a range of 50°C. Error bars represent the mean value of half bandwidths of Gaussian distributions of sliding velocities obtained from statistical analysis of motility time lapse images averaged over 3 to 5 independent experiments for each temperature point. Panel B shows sliding velocities obtained at 20°C as a function of free Mg^2+^-ion concentration. All three data sets were simultaneously fitted using equation 1. Solid lines are the results of the global fit. Thermodynamic parameters of the fits are given in table 1. (C) Kinetic model of the two alternative ADP dissociation steps in the acto-myosin-5b cycle. A is F-actin, M is *Dictyostelium* myosin-5b, Mg^2+^ is the divalent magnesium cation, D is ADP. The top pathway represents the simultaneous dissociation of Mg^2+^ADP and the bottom pathway ADP dissociation from acto-myosin. The two states AM·Mg^2+^D and AMD are in fast equilibrium defined by the dissociation constant *K*_Mg_, which is temperature dependent. (D) Three dimensional surface plot of myosin-5b sliding velocity dependence on temperature and free Mg^2+^-ions calculated from equation 1 using parameters shown in table 1. Experimental points from A and B are included as filled circles.

The motile activity of myosin-5b was studied at two defined free Mg^2+^-ion concentrations [Mg^2+^] over a temperature range of 50°C using *in vitro* motility assays. Figure 1A shows the temperature dependence of the sliding velocity in Arrhenius coordinates and figure 1B depicts the [Mg^2+^] dependence of the sliding velocity at 20°C. The latter data have been reported earlier in the context of the kinetic characterization of the myosin-5b [20]. [Mg^2+^] inhibited actin filament sliding velocity 2.5-fold with an apparent dissociation constant (*K*_Mg_) of 0.4 mM. Thus, saturating free Mg^2+^-ion concentrations do not fully inhibit the motile activity of myosin-5b. Previous kinetic investigations with myosin-5a propose a sequential model of product dissociation in which Mg^2+^ is released prior to ADP [17]. In fact the model is consistent with the observation that [Mg^2+^] inhibit the sliding velocity of the motor [20], but it does not explain why saturating [Mg^2+^] do not completely inhibit sliding velocity. In order to address this problem, we performed temperature-dependent motility assays at two limiting [Mg^2+^] of 0.05 mM and 4.5 mM representative for the high and low limits in sliding velocity at 20°C. Over the entire temperature range velocities at low 0.05 mM [Mg^2+^] (upper trace) were higher than at 4.5 mM [Mg^2+^] (lower trace). For both experiments a non-linear Arrhenius dependence was observed. This indicates that the underlying kinetics, which determine sliding velocity cannot be considered as a single elementary step in the reaction pathway. A similar observation was reported for the sliding velocity driven by muscle myosin-2 [30], [31]. Moreover, such convex non-Arrhenius behavior has been described as characteristic feature of the kinetics for other proteins [32], [33]. Several theoretical approaches have been made to describe this behavior of enzyme catalyzed reactions providing some explanations of non-linearity [34]. Our experimental results imply that the simplest kinetic reaction pathway explaining both non-linear Arrhenius dependence and free Mg^2+^ inhibition should include a fast and temperature sensitive equilibrium between Mg^2+^-bound and Mg^2+^-unbound acto-myosin-ADP (AM**·**D)-states that precede the subsequent and rate limiting steps of the myosin-5b ATPase cycle (Figure 1C). According to this model an alternative pathway in the acto-myosin cycle can be formulated, which includes the simultaneous release of Mg^2+^ and ADP in a single step [16]. Assuming a fast equilibrium between Mg^2+^-bound and Mg^2+^-unbound AM**·**D states, the analytical solution of the proposed kinetic model depicted in Figure 1C can be greatly simplified, where K_Mg_ represents the binding affinity of free Mg^2+^ to AM**·**D and k_1_ and k_2_ correspond to the two rate limiting steps, which determine the dwell times of myosin attached to actin. Presumably, these steps are accompanied by the release of ADP and detachment of myosin from actin. These steps are assumed to be essentially irreversible. The dwell time of the actin attached states of myosin determines the maximum sliding velocity [10]. The analytical solution of the underlying kinetic model gives a rate limiting constant as follows:
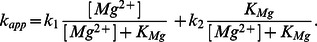
This apparent rate constant determines the overall dwell time of myosin bound to actin. Therefore, the experimentally observed sliding velocity can described as a function of temperature and free Mg^2+^-ion concentrations in the following form:
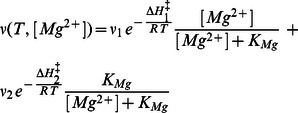
(1)

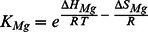
(1′)where R is the universal gas constant, T is the temperature in Kelvin, ΔH_1_^‡^ and ΔH_2_^‡^ are the enthalpies of activation of the two alternative ADP release pathways from acto-myosin, [Mg^2+^] is the concentration of free magnesium ions, *ν*_1_ and *ν*_2_ are pre-exponential factors, which include terms of activation entropy for the corresponding transitions and factors for the conversion of rate-limiting rate constants to the sliding velocity. *K*_Mg_ is the dissociation constant of Mg^2+^ from the AM·Mg^2+^ADP-complex. Equation 1′ represents *K*_Mg_ as a function of standard enthalpy and entropy differences (ΔH_Mg_ and ΔS_Mg_) between Mg^2+^-bound and Mg^2+^-unbound AM·D states.

**Figure 2:**
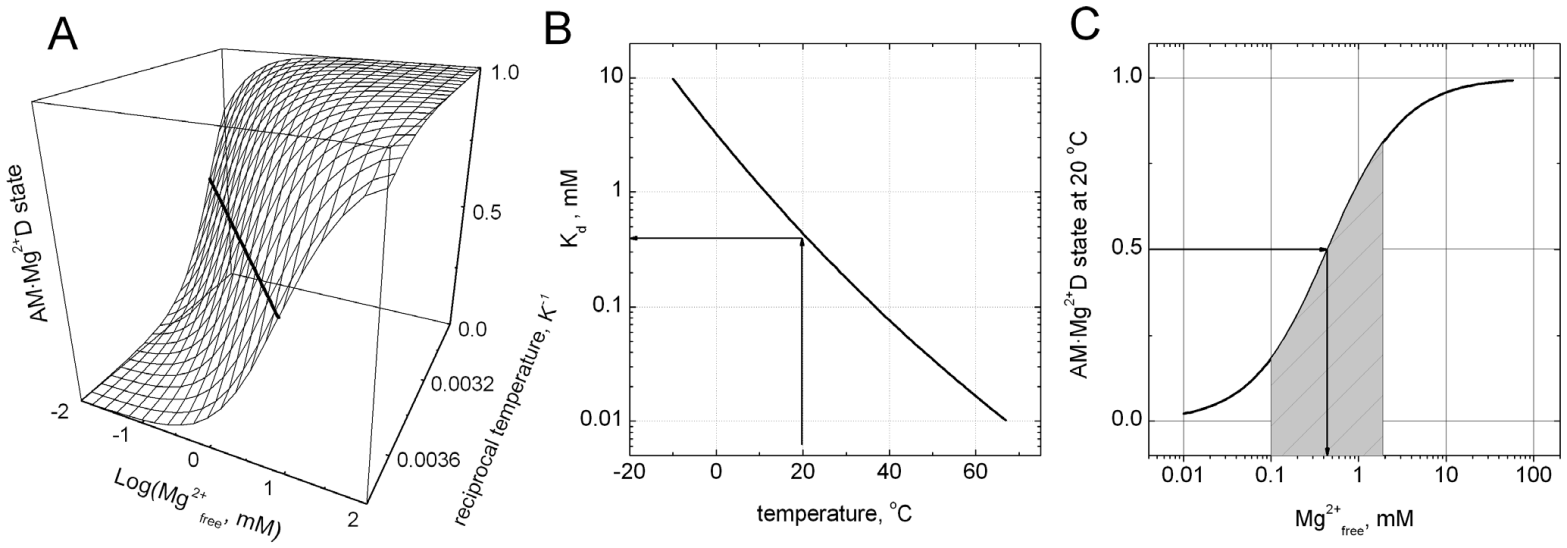
Population of the AM·Mg^2+^D state in the myosin-5b ATPase cycle. Three dimensional surface plot of the fraction of populated AM·Mg^2+^D state 
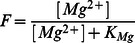
 as a function of temperature and free Mg^2+^-ion concentrations using parameters from table 1. The solid line in the middle of panel A matches *K*_Mg_ of the equilibrium in Figure 1C. The same line as a function of the temperature is shown in panel B. Panel C shows the dependence of F on the concentration of free Mg^2+^ at 20°C. Shadowed area underlines the range of free Mg^2+^-ion concentration where the population of the AM·Mg^2+^D state changes from 0.2 to 0.8.

**Figure 3:**
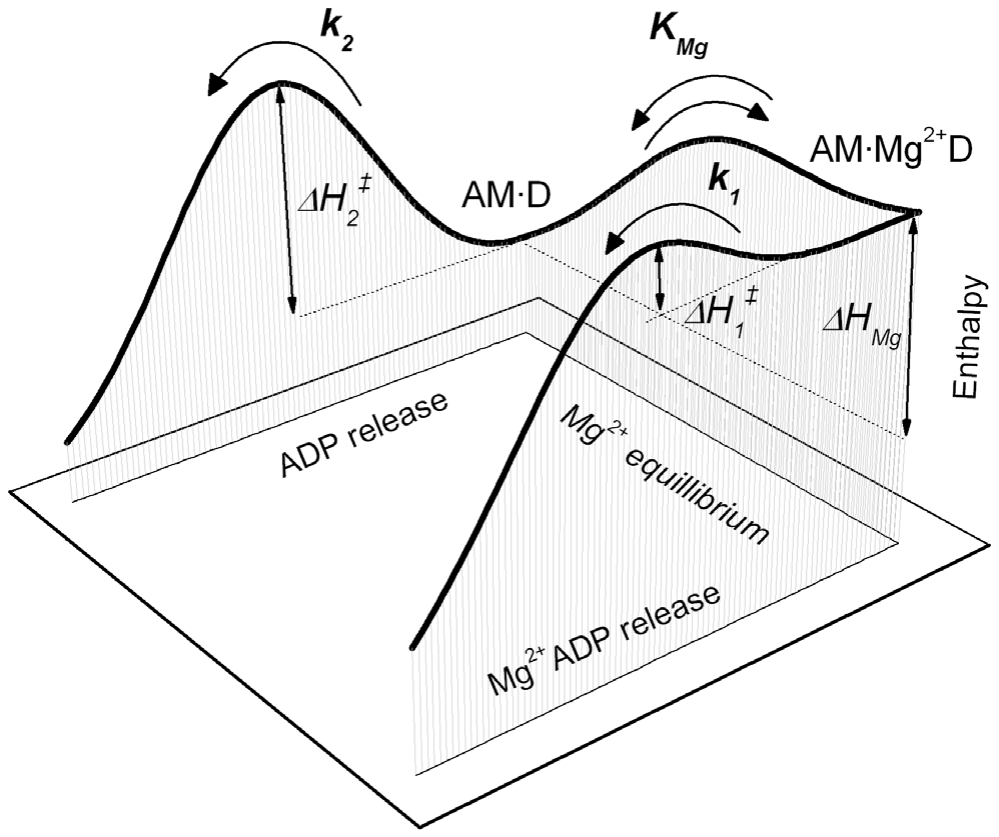
Energy landscape of the Mg^2+^-sensitive states of myosin-5b. Illustrated are the relative changes in enthalpy (ΔH_1_^‡^, ΔH_2_^‡^, ΔH_Mg_) of the reaction according to the proposed kinetic model. The enthalpic barriers ΔH_1_^‡^ and ΔH_2_^‡^ correspond to the two alternative Mg^2+^-ADP and ADP dissociation pathways, respectively. The enthalpy difference of the Mg^2+^-equilibrium ΔH_Mg_ explains the high temperature dependence of the AMD - AM·Mg^2+^D equilibrium and underlines the non-linear behavior of sliding velocity in the Arrhenius plots.

We applied our three data sets (two T-dependences and one free Mg^2+^-concentration dependence) to a global non-linear least square fitting analysis using function 1. Results of the fit are shown as solid lines in Figures 1A,B and as a three-dimensional plot of the observed dependences and the fitting function (Figure 1D). The 3D plot exceeds the range of physiological temperature and concentration of free Mg^2+^-ions occurring in biological systems [35], but illustrates the extent of velocity variation and the highly non-linear character predetermined by the thermodynamic parameters obtained from the fit. These parameters are shown in Table 1. The non-linear Arrhenius behavior can be explained from a temperature-induced shift in the equilibrium between Mg^2+^-unbound and Mg^2+^-bound AM**·**D states. At low temperatures the Mg^2+^-unbound state AM**·**D dominates. The corresponding activation enthalpy ΔH_2_^‡^ of the ADP release is 64 kJ/mol and can be seen from the steepness of the Arrhenius plot in the low temperature range (Figure 1A). At higher temperatures the Mg^2+^-bound AM·Mg^2+^ADP states determines the sliding velocity. This implies that the rate of Mg^2+^-ADP release is slower than the dissociation of ADP alone. On the other hand the activation enthalpy barrier of this pathway ΔH_1_^‡^ is only 18 kJ/mol. This indicates that the entropic part of free energy barrier should be negative and higher in comparison to the entropic part of activation barrier for ADP release without bound Mg^2+^. The activation enthalpy ΔH_1_^‡^ makes the slope in the Arrhenius plot less steep in the high temperature range. The high sensitivity of the Mg^2+^-associated equilibrium (*K*_Mg_) towards the temperature gives a plausible explanation for the convex non-linear behavior of myosin-5b motility in the Arrhenius plots. The pronounced difference in activation enthalpies can be explained as a result of neutralization of the electrostatic charge of ADP by Mg^2+^ and therefore weakening of binding energy to the protein. The 3-fold reduction of activation enthalpy upon binding of Mg^2+^ is in line with an increase in the enthalpy (ΔH_Mg_) of the Mg^2+^-bound state of the equilibrium. The equilibrium shift towards the AM**·**Mg^2+^D at higher temperatures implies that this state is entropically favored (ΔS_Mg_ = +234 J/molK). Moreover, since the AM**·**D state has a lower entropy than the AM**·**Mg^2+^D state, we can assume that the height of the entropic barrier for the release of Mg^2+^-ADP is dictated by the entropy of the AM**·**Mg^2+^D state. As a consequence, the rate constant for Mg^2+^-ADP release becomes slower. From the *v*_1_ and *v*_2_ pre-exponential factors we can estimate the difference of entropic activation barriers between the two pathways: 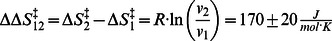. The uncertainty of the obtained value was propagated from the errors of ν_1_ and ν_2_ (see Table 1). This estimation clearly indicates that the contribution of ΔS_Mg_ to the difference in activation entropies is significant. To show the range by which [Mg^2+^] and temperature influence the equilibrium we plotted the corresponding fraction of the AM**·**Mg^2+^D state 
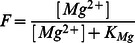
 in a 3D graph shown in figure 2A. The solid line in the surface plot matches the value *F* = 0.5, which defines *K*_Mg_. Figure 2B depicts this line as a function of the temperature. According to this plot, *K*_Mg_ decreases 90-fold from 1.8 to 0.02 mM in the experimental temperature range (5 to 55°C). The high enthalpy difference ΔH_Mg_ of 66.5 kJ/mol accounts for the large change in *K*_Mg_. Note, in aqueous solution (pH 7.0) the increase in enthalpy for Mg^2+^ binding to ADP is approximately 10.5 kJ/mol, as calculated from the temperature dependence of the dissociation constant of the Mg^2+^-ADP complex according to NIST database parameters of the Maxchelator program. This calculation indicates that the process of Mg^2+^-ion coordination in the nucleotide binding site of myosin-5 is accompanied by a significant change in the energy of interactions by the protein surrounding. On the other hand the entropy driven mechanism of binding does not principally change (according to NIST database the Mg^2+^-ADP complex at pH 7.0 in aqueous solution has a 95 J/molK higher standard entropy than the dissociated form). The fraction of the AM·Mg^2+^D state depends on the concentration of free Mg^2+^-ions. This is shown exemplary for 20°C in figure 2C. Most pronounced variation of the equilibrium occurs around the inflection point of the sigmoidal curve, which covers the range of free Mg^2+^-ion concentration from approximately 0.1 to 2 mM. Interestingly, this concentration range matches the physiological range of free Mg^2+^-ions found in cells [36]. Therefore, the observed changes in the spatial and temporal distribution of free Mg^2+^-ions might be of importance for regulation of myosin motor function *in vivo*. Figure 3 shows the energetic landscape of the Mg^2+^-regulated part of the enzymatic relaxational pathway obtained from our data analysis. The diagram illustrates the temperature independent enthalpic difference of the Mg^2+^-equilibrium and two enthalpic barriers of the rate limiting steps of the reaction. Obviously, the actual rates of transitions and population of states within the equilibrium are defined by the Gibbs free energy values (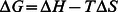) and therefore temperature dependent. A parameter not defined in this scheme is the activation enthalpy barrier of the equilibrium between the AM·Mg^2+^D and AM·D states. This barrier is drawn low enough to be consistent with the model assumption of a fast equilibrium.

**Table 1:** Thermodynamic parameters of the two alternative ADP-release pathways in acto-myosin-5b.

Parameter	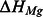 (kJ mol^−1^)	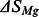 (J mol^−1^ K)^a^	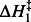 (kJ mol^−1^)	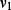 (µm s^−1^)	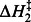 (kJ mol^−1^)	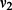 (µm s^−1^)
**Value ± error**	66.5±7.2	234±25	18±3	590±600	64±7	4×10^11^±1×10^12^

Note: ΔS_Mg_ is by the value of 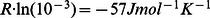 smaller than the standard entropy difference (ΔS_Mg_^0^) because Mg^2+^-ion concentrations have been used in mM units for data analysis.

Our kinetic and thermodynamic analysis of myosin-5b allowed us to describe the Mg^2+^-dependence of motile activity and regulation of this motor by a simple two-state kinetic model. The herein reported methodological approach of data analysis by global fitting can be useful to interpret kinetic and thermodynamic data of other experimentally accessible reaction steps in the acto-myosin ATPase cycle, thus providing a consistent description of the energetics and equilibria of acto-myosin interactions. Moreover, our results underline the significant contribution of entropic terms to the free energies for acto-myosin interactions. At least four additional myosins including *Dictyostelium* myosin-1D and -1E, human nonmuscle myosin-2c, and human myosin-7a show similar variations in their kinetics as reported for myosin-5a and myosin-5b, with the ADP release step being the rate limiting and Mg^2+^-ion sensitive step in their ATPase cycle [37], [38], [39]. The examination of the equilibria states of these myosins can provide further validation of our model assumption.
